# Quercetin and Tranylcypromine Improve Memory, Behavioral Performance, and Cholinergic Function in Male Rats Subjected to Chronic Restraint Stress

**DOI:** 10.3390/brainsci15070709

**Published:** 2025-07-01

**Authors:** Vitor Bastianello Mostardeiro, Charles Elias Assmann, Adriel Antonio Schirmann, Marcylene Vieira da Silveira, Bianca Vedoin Copês Rambo, Mairin Schott, Aline da Silva Pereira, Vanessa Valéria Miron, Heloiza Winck Soares, Larissa Varotto Dambrós, Sabrina Fontana Belinazo, Thamires Gelatti Vidal, Margarete Dulce Bagatini, Maria Rosa Chitolina Schetinger, Vera Maria Melchiors Morsch

**Affiliations:** 1 Graduate Program in Biological Sciences: Toxicological Biochemistry, Department of Biochemistry and Molecular Biology, Center of Natural and Exact Sciences, Federal University of Santa Maria (UFSM), Santa Maria 97105-900, RS, Brazil; charles.assmann@ufsm.br (C.E.A.); adriel.schirmann@acad.ufsm.br (A.A.S.); marcylene.vieira@acad.ufsm.br (M.V.d.S.); biancarambo@outlook.com (B.V.C.R.); mairinschottms@gmail.com (M.S.); aline.s_pereira@hotmail.com (A.d.S.P.); vanessavmiron@gmail.com (V.V.M.); heloizawincksoares@gmail.com (H.W.S.); larissadambros@hotmail.com (L.V.D.); sfontanabelinazo@gmail.com (S.F.B.); thgelatti@gmail.com (T.G.V.); mariachitolina@gmail.com (M.R.C.S.); veramorsch@gmail.com (V.M.M.M.); 2 Graduate Program in Biomedical Sciences, Federal University of Fronteira Sul (UFFS), Chapecó 89815-899, SC, Brazil; margarete.bagatini@uffs.edu.br

**Keywords:** major depressive disorder (MDD), antidepressants, chronic stress, anxiety, natural compounds, acetylcholinesterase (AChE), gene expression, Western blotting

## Abstract

**Background/Objectives**: Major depressive disorder (MDD) is a debilitating illness, and chronic stress is a contributing factor for depressive symptoms. However, despite intense research, the mechanisms of MDD remain substantially unidentified. Quercetin is a powerful flavonoid and could be used as a possible therapeutic strategy for depression. Acknowledging the potential benefits of quercetin, this study investigated its effect alone or in association with the standard drug tranylcypromine (TCP) in a rodent model of chronic restraint stress (CRS). **Methods**: Adult male rats were subjected to a CRS model consisting of an immobilization session of 4 h daily during 14 consecutive days. Quercetin (50 mg/kg, gavage) was administered for 45 days. TCP (10 mg/kg, gavage) was administered for 14 days. Behavioral tasks were conducted to assess locomotor functions, memory, anhedonia, depression-like behaviors, and anxiety-like behaviors. The activity, gene expression, and protein density of acetylcholinesterase (AChE) were investigated. **Results**: Behavioral tasks showed that the CRS model effectively induced stable behavioral changes. CRS did not alter locomotor function assessed by the open field test (OFT) or anhedonia behavior assessed by the sucrose preference test (SPT). CRS increased total fecal count, which was prevented by quercetin administration in rats. TCP and the association of quercetin and TCP increased the recognition index in comparison with the CRS group in the novel object recognition (NOR) test and improved the swimming and immobility times in comparison to stressed animals in the forced swim test (FST). All treatments were able to decrease the anxiety index assessed by the elevated plus maze (EPM) test. The activity, gene expression, and protein density of AChE were increased in the CRS model compared to control males. Overall, quercetin and TCP proved to reverse CRS-induced alterations in these parameters. **Conclusions**: Quercetin mitigated cognitive deficits, behavioral impairments, and neurochemical alterations induced by the CRS model, especially in association with TCP, supporting its potential as a promising therapeutic agent for depression.

## 1. Introduction

Major depressive disorder (MDD) or depression is a severe mood disorder affecting almost 300 million people worldwide [1]. It is characterized by a complex interplay of several physical and emotional symptoms, such as depressed mood, feelings of sadness and loneliness, fatigue, anxiety, lack of motivation, cognitive impairment, loss of pleasure or interest in activities (referred to as anhedonia), alterations in appetite and weight, and sleep disturbances, among others that impose a significant burden to affected individuals [1,2,3].

MDD is a multifaceted psychiatric illness. Biological, genetic, psychological, environmental, and social factors are thought to participate in its etiology [4]. Neurobiological factors such as cholinergic dysfunctions may play a considerable role in the cognitive impairment [5] and depressed mood [6] observed in patients. Structural and functional alterations in brain areas and circuits, like cholinergic signaling, may also arrive due to factors such as chronic stress. It has been postulated that prolonged and unpredictable stress predisposes individuals to the development of depression [5,7]. Therefore, chronic restraint stress (CRS) is extensively employed in rodent animal models to induce a depression-like phenotype [8]. However, the neurobiological pathways involved in this complex disease are far from being completely understood.

Several treatment options are currently available for depression, including psychological therapies and pharmacological interventions [1]. As one of the main hypotheses for depression is the functional deficiency of monoamines (serotonin, norepinephrine, and dopamine) in the brain, monoamine-based antidepressants such as Serotonin Reuptake Inhibitors (SRI), Norepinephrine Reuptake Inhibitors (NRI), and Monoamine Oxidase Inhibitors (MAOI) are prescribed in the clinic. Through distinct mechanisms, these drugs are used to increase the availability of monoamines in the brain ameliorating symptoms of the disorder [9,10]. Non-selective MAOIs like tranylcypromine (TCP), also known as *trans*-2-phenylcyclopropylamine (2-PCPA), influence monoaminergic neurotransmission and have been used clinically for the treatment of MDD [11,12]. However, antidepressants may present side effects and non-pharmacological approaches may benefit patients and improve the action of medications, also reducing undesirable outcomes.

Evidence has shown that some molecules, such as the flavonoid quercetin, may be able to influence the levels of monoamines in the brain [13,14,15]. Serotonin (5-hydroxytryptamine; 5-HT) is preferentially oxidized by monoamine oxidase A (MAO-A) and quercetin was shown to attenuate the activity of this enzyme in the brain [16,17]. Quercetin is naturally found in several food sources such as caper (234 mg/100 g), radish (70 mg/100 g), and onion (32 mg/100 g) [18], and its daily intake is estimated to be around 5–40 mg [19]. Quercetin has been shown to present several biological properties, including antioxidant, anti-inflammatory, and anti-cancer properties [20], and its role as an antidepressant-like molecule has been suggested by the literature [19,21]. Research has also highlighted the prominent role of quercetin in improving memory potentially by the modulation of cholinergic pathways [22,23]. However, despite research on the role of quercetin in depression, the potential underlying protective mechanisms are far being completely understood.

Based on this background, the aim of this study was to investigate the protective role of quercetin and its possible synergistic action mode with tranylcypromine (TCP) in a rodent model of depression-like behavior. Animals were subjected to a CRS model and a set of behavioral tasks were conducted to assess locomotor functions, memory, anhedonia, anxiety-like and depression-like behaviors in male rats. The changes in cholinergic function, mainly acetylcholinesterase (AChE) enzyme, were also explored.

## 2. Materials and Methods

### 2.1. Chemicals, Reagents, and Equipment

Quercetin was acquired from Sigma-Aldrich (St. Louis, MO, USA). Tranylcypromine (TCP) (GlaxoSmithKline Ltd., Rio de Janeiro, RJ, Brazil) was purchased from a local pharmacy (Santa Maria, Brazil). All other reagents were obtained from Sigma-Aldrich or Merck KGaA (Darmstadt, Germany) or otherwise not stated were of the highest purity. Reagents for molecular assays were purchased from ThermoFisher Scientific (New York, NY, USA), Santa Cruz Biotechnology (Dallas, TX, USA), and Bio-Rad Laboratories (Hercules, CA, USA). The spectrophotometric measurements were carried out using a SpectraMax^®^ i3 Multimode Plate Reader (Molecular Devices, Sunnyvale, CA, USA). The imaging of gels and blots was performed using the ChemiDoc MP Imaging System (Bio-Rad Laboratories, Hercules, CA, USA). Gene expression was performed using the Applied Biosystems QuantStudio™ 3 Real-Time PCR System (ThermoFisher Scientific, New York, NY, USA).

### 2.2. Animals and Ethical Approval

Adult male Wistar rats (n  =  60; 50–60 days old; weight 200–250 g) obtained from the UFSM (Santa Maria, Brazil) Animal Reproduction Center were used in the experiments. The number of animals was calculated by the G*Power software (ver. 3.1.9.7; Heinrich-Heine-Universität Düsseldorf, Düsseldorf, Germany) resulting in a sample size of 60 experimental units. Animals were housed in polypropylene cages measuring 18 cm in height, 34 cm in width, and 48 cm in length. They were kept under standardized conditions of temperature (22  ±  1 °C), humidity (55  ±  10%), filtered air, and a 12 h light/dark cycle, with lights turned on at 7 am. Filtered water and commercial food were provided freely, except during the periods of CRS protocols.

The same researchers conducted all treatments and behavioral assays during the same time periods each day. All efforts were made to reduce the number of animals used in the protocols to a minimum. All procedures strictly adhered to ethical requirements and were approved by the Ethics Committee on Animal Research (CEUA) of the UFSM (Approval Code CEUA No 3315241022; Approval date 22 November 2022) and carried out in accordance with the recommendations in the Guide for the Care and Use of Laboratory Animals of the National Institutes of Health (NIH).

### 2.3. Experimental Protocol

#### 2.3.1. Chronic Restraint Stress (CRS) Model

A chronic restraint stress (CRS) model was established according to previous protocols [24,25]. The rats were immobilized without food and water in an individual plastic apparatus (25 cm length × 7 cm diameter) with holes (1 cm in diameter) to allow respiration. The apparatus did not allow the animals to move freely or turn around, but they were not oversqueezed to avoid pain or injury. At the end of each session, the animals were immediately returned to their home cages. The protocol of the CRS model consisted of an immobilization session of 4 h daily (1 pm to 5 pm) during 14 consecutive days, known to induce stable behavioral changes that closely resemble a depression-like phenotype [24]. The animals of the control group (non-stressed) and quercetin (non-stressed) were left undisturbed in their home environment at the time of the CRS, without food and water. The apparatus was thoroughly cleaned with an ethanol solution (70% *v*/*v*) and dried with paper towels after each behavioral session.

#### 2.3.2. Treatments

Quercetin at the dose of 50 mg/kg [26,27,28] was dissolved in Tween 3% (vehicle) and administered via gavage for 45 consecutive days. Tranylcypromine (TCP) at the dose of 10 mg/kg [12,29,30] was dissolved in the vehicle and administered orally. The animals of the control (non-stressed) and CRS (subjected to stress) groups received only the vehicle via gavage. Treatments were prepared immediately prior to administration. The animals of each group received their respective treatments once daily at the same time (9 am to 12 am) by the same researchers.

#### 2.3.3. Experimental Design

**Figure 1:**
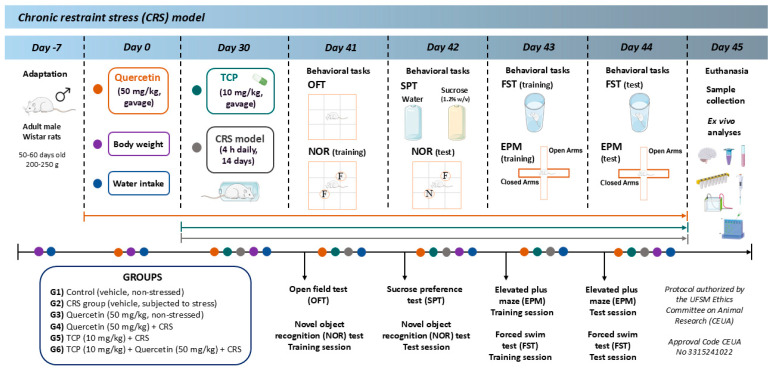
Representative image of the experimental study design. Following the 7-day adaptation phase, animals were randomly allocated into 6 experimental groups: (**1**) Control (vehicle, non-stressed); (**2**) CRS group (vehicle, subjected to stress); (**3**) Quercetin (50 mg/kg, non-stressed); (**4**) Quercetin (50 mg/kg) + CRS; (**5**) TCP (10 mg/kg) + CRS; (**6**) TCP (10 mg/kg) + Quercetin (50 mg/kg) + CRS. Quercetin and TCP were administered via oral gavage. Treatments were prepared freshly before each administration. The CRS groups were subjected to the CRS protocol for 4 h daily during the 14 consecutive days. The control (non-stressed) and quercetin (non-stressed) groups were left undisturbed in their home cages at the time of the CRS protocol, without food and water. The body weight and water intake were measured throughout the experimental protocol. The following behavioral tasks were conducted: sucrose preference test (SPT), open field test (OFT), novel object recognition (NOR) test, elevated plus maze (EPM), and forced swim test (FST). The animals underwent euthanasia 24 h after the completion of the behavioral tasks and the samples were collected for biochemical and molecular analysis. CRS: chronic restrained stress; TCP: tranylcypromine; Vehicle: Tween 3%. (This figure was created by using templates from https://smart.servier.com).

Before the beginning of the experiments, the animals underwent a 7-day adaptation step to the housing conditions. The animals were randomly allocated in the following groups (n = 10 per group): (1) Control (vehicle, non-stressed); (2) CRS group (vehicle, subjected to stress); (3) Quercetin (50 mg/kg, non-stressed); (4) Quercetin (50 mg/kg) + CRS; (5) TCP (10 mg/kg) + CRS; (6) TCP (10 mg/kg) + Quercetin (50 mg/kg) + CRS. After the adaptation period, quercetin was administered everyday throughout the whole experimental period. After 30 days, the CRS model was conducted for 14 consecutive days. On the same day, the administration of TCP was also initiated, during the 14 consecutive days. In the last days of the experimental protocol, a set of behavioral tasks was conducted to assess locomotor functions, memory, anhedonia, anxiety-like, and depression-like behaviors. Afterwards, 24 h after the final behavioral tasks, the animals were anesthetized under a 5% isoflurane atmosphere and then euthanized. The experimental timeline of the study is illustrated in Figure 1.

### 2.4. Sample Collection

Brain samples were obtained from the rats, and the cerebral cortex and hippocampus structures were carefully excised on ice. Each sample was rinsed with cold saline solution (0.9% NaCl) and immediately stored in freezer tubes and placed at −80 °C for long-term preservation. Upon thawing, samples were homogenized in specific buffers according to the requirements of each assay.

### 2.5. Body Weight and Water Consumption

During the experimental period, the weight of each animal was measured twice a week. Body weight change was calculated by the percentage of change in the body weight in relation to the previous measurement. The water intake of each box was measured daily and averaged over three days for clearer visualization. The mean consumption of water per animal was calculated as the total consumption of the cage/number of animals per cage.

### 2.6. Behavioral Tasks

#### 2.6.1. Sucrose Preference Test (SPT)

The sucrose preference test (SPT) was conducted to assess the anhedonia behavior of rats [25]. For this, animals were allocated into cages with two identical weighed bottles containing only filtered water. During the task, commercial food was provided ad libitum. After 4 h of adaptation, one of the bottles was randomly changed by another containing a 1.2% (*w*/*v*) sucrose solution. The total intake of water and sucrose solution of each cage was measured after 16 h. The sucrose percentage preference was calculated as the percentage of consumed sucrose solution relative to total liquid consumption [25].

#### 2.6.2. Open Field Test (OFT)

The open field test (OFT) was conducted to investigate the locomotor and exploratory behavior of rats [31]. The animals performed the OFT in a box measuring 1 m × 1 m with nine equal divisions. The animals were placed individually in the middle of the arena and allowed to move freely. The test lasted 10 min, and the number of crossings in the center, grooming time, total distance, total fecal count, and number of rearings were measured. The protocol was recorded with a camera, and the ANY-maze™ (version 7.44, Stoelting CO, Wood Dale, IL, USA) software was employed to perform the analysis of specific locomotor parameters. The apparatus was thoroughly cleaned with an ethanol solution (70% *v*/*v*) and dried with paper towels before each behavioral trial [31].

#### 2.6.3. Novel Object Recognition (NOR) Test

The novel object recognition (NOR) test was used to explore the neurobiology of memory in rodents subjected to the CRS model [32,33]. The task was conducted in two steps (2 consecutive days) and occurred 24 h following the OFT within the same apparatus, which also served as the habituation session for the NOR test. In the first step (NOR training), two odorless and identical objects (size, color, and shape) were placed equally distant in the apparatus, and the animals were allowed to explore them freely for 5 min. After 24 h, in the second step (NOR test), one of the objects was replaced with a novel object (N), which was different in size, color, and shape from the familiar object (F), and animals were allowed to explore both objects for 5 min [32,33]. The recognition index was then calculated considering the difference between the time spent exploring the novel (N) and the familiar (F) object × 100 divided by the total time spent exploring the novel (B) and the familiar (A) objects and used as a cognitive parameter ([(Tnovel − Tfamiliar)/(Tnovel + Tfamiliar)]/100). The apparatus and objects were thoroughly cleaned with an ethanol solution (70% *v*/*v*) and dried with paper towels before each behavioral trial. The protocol was recorded with a camera and later analyzed using the ANY-maze™ (version 7.44, Stoelting CO, Illinois, USA) software.

#### 2.6.4. Elevated Plus Maze (EPM)

The elevated plus maze (EPM) test was used to assess the anxiety-like behavior in rats [34] in 2 consecutive days (training and test sessions). The apparatus consisted of 4 arms of equal dimensions, 2 of them were closed and 2 were open. Briefly, the animal was positioned in the center of the apparatus facing a closed arm and allowed to freely explore the arms for 8 min. The task included 2 min of adaptation and 6 min of testing. An entry was defined as placing all four paws within the open/closed arm. The sequence and number of entries in each arm was calculated by the following formula: percentage time spent in the open arms (OAT% = [time spent in the open arms/total time spent in the arms] × 100); percentage of entries in the open arms (OAE% = [number of entries in the open arms/number of total entries] × 100). The anxiety index was calculated by 1 − [([time in the open arms/duration of the test session] + [entries in the open arms/number of total entries])/2] [35]. The apparatus was thoroughly cleaned with an ethanol solution (70% *v*/*v*) and dried with paper towels before each behavioral trial. The total duration of each trial was recorded with a camera, and the parameters were analyzed using the ANY-maze™ (version 7.44, Stoelting CO, Wood Dale, IL, USA) software.

#### 2.6.5. Forced Swim Test (FST)

The forced swim test (FST) was used to assess depression-like behavior in rodents based on the immobility time of animals. An increase in the immobility time is indicative of a depression-like effect [36,37]. This protocol was conducted over 2 consecutive days, which consisted of a pre-swimming session and a test session. Briefly, rats were individually placed in an open and opaque cylindrical container (50 cm height × 20 cm diameter) filled with a 25 cm column of water at 25 ± 1 °C and forced to swim during 6 min. In the test session (24 h after the pre-swimming session), swimming and immobility durations were recorded during a 6 min period. After the sessions, the rats were gently dried and heated by a lamp in a box covered by sterile wood shavings before being returned to their home cages. Swimming was described as movements through the water column, while immobility referred to the animal floating motionless or making slight movements to keep its head above the water. Swimming duration was calculated as the total test duration − immobility time [25]. The water was completely changed before each trial; the apparatus was also thoroughly cleaned, then sprayed with an ethanol solution (70% *v*/*v*) and dried with paper towels.

### 2.7. Enzymatic Assays

#### Acetylcholinesterase (AChE) Enzymatic Activity

AChE activity in the cerebral cortex and hippocampus was performed as described before [38]. Initially, samples were homogenized on ice with 10 mM Tris-HCl buffer (1:10 ratio), then centrifuged at 1800 rpm for 10 min, and the supernatant was collected for the assay. The reaction mixture consisted of 15 µL of each brain structure, 100 mM phosphate buffer (pH 7.5), and 10 mM 5,5′-Dithiobis(2-nitrobenzoic acid) (DTNB). The 96-well plate was pre-incubated at 25 °C for 2 min before adding the substrate, 8 mM acetylthiocholine iodide (AChI). Then, the enzymatic reaction was monitored for 2 min at 412 nm. The enzyme activity was expressed as ƞmol ACh hydrolyzed/min/mg of protein. In this assay, the protein content of samples was determined and normalized using the Coomassie Brilliant Blue method with albumin as the standard [39].

### 2.8. Molecular Assays

#### 2.8.1. Quantitative Real-Time Polymerase Chain Reaction (qRT-PCR)

Gene expression of AChE in the cerebral cortex and hippocampus tissues was analyzed by quantitative real-time polymerase chain reaction (qRT-PCR), similarly to what was published before [40]. After RNA extraction and quantification at 260 nm, cDNA was obtained by reverse transcription using a cDNA synthesis kit according to the manufacturer’s instructions. The molecular assay was carried out using a SYBR^®^ Green PCR Kit and the cDNA samples. Gene expression analysis was conducted using the 2^−ΔΔCT^ method, with β-Actin as the reference gene for normalization. The sequences of forward (F; 5′-3′) and reverse (R; 5′-3′) primers were as follows: β-Actin F (TGTGACGTTGACATCCGTAAAG) and R (GGCAGTAATCTCCTTCTGCATC); AChE F (GAATCTTTGCTCAGCGACTTATG) and R (AGGTTCAGGCTCACGTATTG).

#### 2.8.2. Immunoblotting

Protein levels of AChE were assessed by Western blotting, incorporating modifications based on a previous study [41]. Samples were initially homogenized in radioimmunoprecipitation assay (RIPA) extraction buffer with protease inhibitors and protein content was measured by the bicinchoninic acid (BCA) assay. Then, 30 μg of the total protein and protein marker were loaded and separated on an SDS-polyacrylamide gel by electrophoresis. Proteins were transferred to nitrocellulose membranes and blocked with 3% bovine serum albumin (BSA) in TBS with 0.1% Tween-20 for 1 h. Subsequently, the membranes were incubated overnight at 4 °C with specific primary antibodies. The dilutions of primary antibody were 1:200 for anti-AChE (SC-6432, Santa Cruz Biotechnology) and 1:5000 for anti-β-Actin (A-2228, Sigma-Aldrich). After washing with a TBS solution containing 0.1% Tween-20, the membranes were incubated with an appropriate secondary antibody (1:10,000, ThermoFisher Scientific) for 2 h at room temperature. The immune-reactive proteins were detected using enhanced chemiluminescence (ECL) Western blotting Immobilon Forte Western HRP substrate (Merck KGaA). Results were shown as the fold change relative to β-Actin.

### 2.9. Statistical Analysis

The results are presented as mean ± standard error of the mean (S.E.M.), derived from a minimum of three different experiments. Statistical comparisons were carried out by one-way analysis of variance (ANOVA), and Bonferroni’s post hoc test was utilized for multiple comparisons between groups. Statistical analyses were performed using the GraphPad Prism 9.0 software (San Diego, CA, USA). A *p*-value of <0.05 was set to indicate statistically significant results.

## 3. Results

### 3.1. Alterations in Body Weight, Water Consumption, and Sucrose Preference in Animals Subjected to CRS

**Figure 2:**
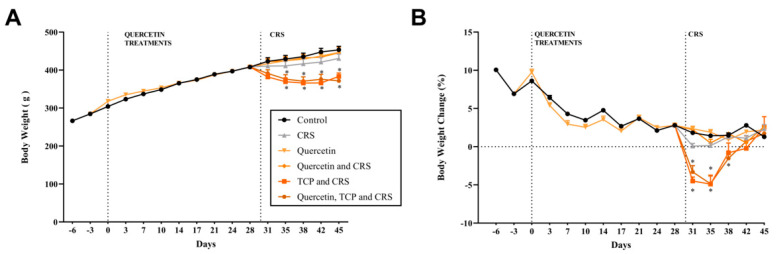
In vivo effects of treatments with Quercetin (50 mg/kg) and Tranylcypromine (10 mg/kg) on the weight of animals subjected to CRS. (**A**) Body weight in grams. (**B**) Percentage of body weight variation in relation to the previous measurement. Control = non-stressed. CRS = chronic restraint stress; TCP = tranylcypromine. Values are expressed as mean ± standard error of the mean (n = 10). * Represents statistical significance in relation to the control (non-stressed) group (*p* < 0.05). One-way ANOVA followed by Bonferroni post hoc test.

**Figure 3:**
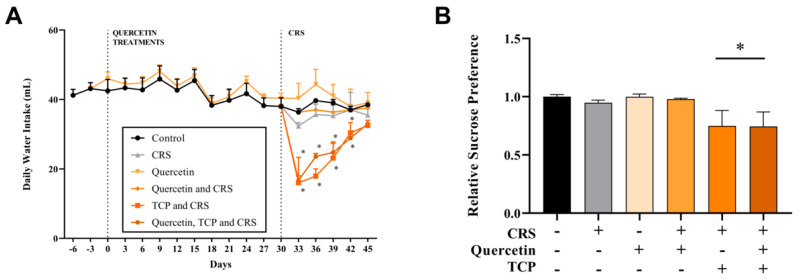
In vivo effects of treatments with Quercetin (50 mg/kg) and Tranylcypromine (10 mg/kg) on water consumption and sucrose preference of animals subjected to restraint stress. (**A**) Average daily water consumption (in mL) per animal. (**B**) Relative preference for sucrose in relation to the negative control group. Control = non-stressed. CRS = chronic restraint stress; TCP = tranylcypromine. Values are expressed as mean ± standard error of the mean (n = 4). * Represents statistical significance in relation to the control (non-stressed) group (*p* < 0.05). One-way ANOVA followed by Bonferroni post hoc test.

During the maintenance of the animals in their housing conditions, body weight (Figure 2A,B) was measured biweekly. Water consumption (Figure 3A) and overall well-being of the animals were monitored daily. After a 7-day adaptation period, the administration of quercetin was initiated (day zero). After 30 days of treatment, the administration with TCP and the CRS model were started in the respective groups, until completing the 45 days of the experimental protocol. This study aimed to explore the effects of interventions on various physiological and behavioral parameters using established experimental models. Body weight, water intake, and sucrose preference, often key indicators of stress-induced changes, were meticulously monitored alongside advanced molecular and protein assays.

Figure 2A,B show the alterations in the body weight during the experimental protocol. No significant changes in the body weight were observed between groups from the start of the adaptation phase to the beginning of the CRS protocol and TCP treatment (*p* > 0.9999). The administration of quercetin from day 0 until day 30 also did not alter body weight (*p* > 0.9999). However, the groups subjected to the CRS model and that received the administration of TCP alone or in association with quercetin presented a significant reduction in the body weight from day 35 until the end of the experiment in comparison to the control group (F_5, 54_ = 2.47; *p* = 0.0081 and *p* = 0.0044 for day 35; *p* = 0.0003 and *p* = 0.0086 for day 38; *p* < 0.0001 and *p* = 0.0183 for day 42; *p* = 0.0155 and *p* = 0.0007 for day 45, respectively). The group subjected to the stress model, but not receiving any intervention, showed a tendency to decrease this parameter; however, this change was not statistically significant (*p* > 0.9999) (Figure 2A).

Figure 2B demonstrates the percentage of body weight variation in relation to the previous measurement. The results show that animals subjected to the stress protocol and that received TCP or TCP in association with quercetin significantly decreased this parameter beginning from day 31 in comparison to the control group (F_5, 834_ = 15.80; *p* < 0.0001 and *p* = 0.0012 for day 31; *p* = 0.0073 and *p* = 0.0033 for day 35, respectively; *p* = 0.0436 for day 38). However, after day 38, animals subjected to the stress protocol and that received TCP or TCP in association with quercetin returned this parameter to control group levels (*p* = 0.9163 and *p* = 0.9916, respectively) (Figure 2B).

Figure 3A demonstrates the water intake of each box (represented as the mean consumption of three days). Results show that from the adaptation step until the beginning of the administration of TCP and stress model no significant changes in water consumption between groups were observed (F_5, 18_ = 1.46; *p* > 0.05). At day 33 until day 42, a significant reduction in water intake was observed for the groups subjected to the stress model and that received the administration of TCP or its association with quercetin in comparison to the control group (F_5, 18_ = 1.46; *p* = 0.0046 and *p* = 0.0001 for day 33; *p* < 0.0001 and *p* = 0.0039 for day 36; *p* = 0.0003 and *p* = 0.0011 for day 39; *p* = 0.0329 and *p* = 0.0487 for day 42, respectively). This decline in water intake for these groups returned to control levels during the final 3 days of the experimental protocol (*p* > 0.9999 and *p* = 0.3139, respectively) (Figure 3A).

In the final days of the experimental protocol, the animals underwent a series of tasks to assess depression-like behavior. The sucrose preference test (SPT) was conducted to assess the anhedonia behavior of the animals (Figure 3B). The results demonstrate that animals subjected to the stress model and that received TCP intervention or its association with quercetin significantly decreased the relative sucrose preference in comparison to control (non-stressed) animals (F_5, 18_ = 10.50; *p* = 0.0022 and *p* = 0.0018, respectively). Animals subjected to the stress protocol showed no significant differences compared to control (non-stressed) animals (*p* = 0.9227) (Figure 3B).

### 3.2. Quercetin and TCP Interventions Improve Memory, Depression-like, and Anxiety-like Behaviors in Male Rats Subjected to CRS

**Figure 4:**
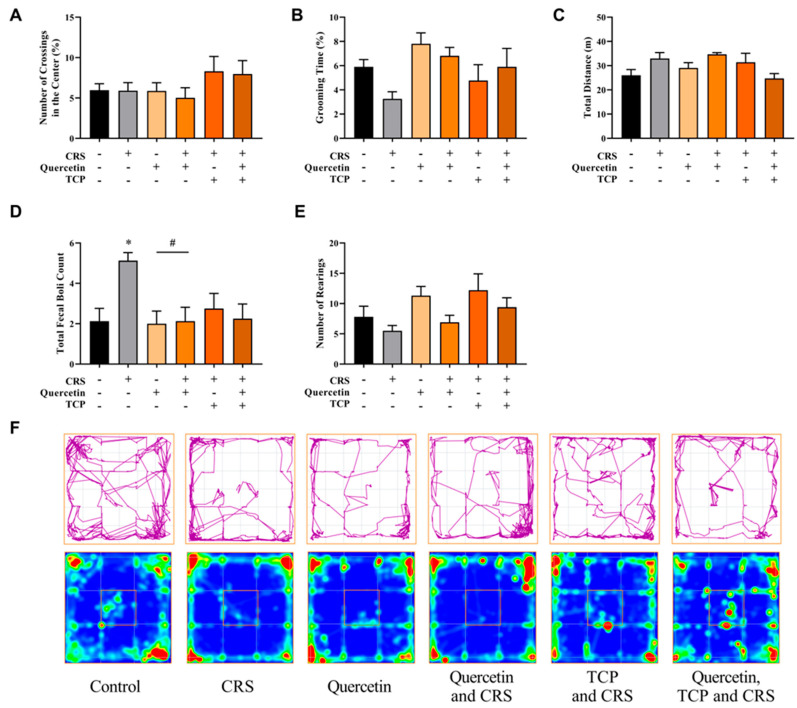
In vivo effects of treatments with Quercetin (50 mg/kg) and Tranylcypromine (10 mg/kg) in animals subjected to restraint stress in the open field test (OFT). (**A**) Percentage number of crossings in the central zone of the OFT apparatus. (**B**) Percentage of time spent on self-grooming. (**C**) Total distance (m) traveled. (**D**) Total number of fecal boluses (fecal output) during the test. (**E**) Number of rearings. (**F**) Representative tracking plots of an animal of each experimental group (first row). Heat map plots are representative of the mean of each group (second row). CRS = chronic restraint stress; TCP = tranylcypromine. Values are expressed as mean ± standard error of the mean (n = 8). * Represents statistical significance in relation to the control (non-stressed) group (*p* < 0.05). # Represents statistical significance in relation to the CRS group (*p* < 0.05). One-way ANOVA followed by Bonferroni post hoc test.

**Figure 5:**
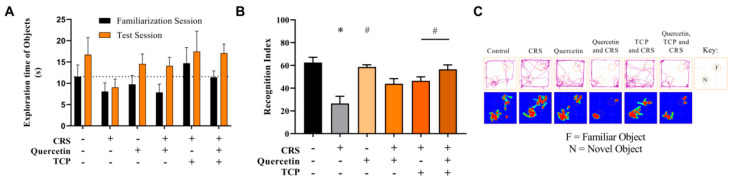
In vivo effects of treatments with Quercetin (50 mg/kg) and Tranylcypromine (10 mg/kg) in animals subjected to restraint stress in the novel object recognition (NOR) test. (**A**) Exploration time (s) of objects in the training and test sessions. (**B**) Recognition index. (**C**) Representative tracking plots of an animal of each experimental group (first row). Heat map plots are representative of the mean of each group (second row). CRS = chronic restraint stress; TCP = tranylcypromine. Values are expressed as mean ± standard error of the mean (n = 8). * Represents statistical significance in relation to the control (non-stressed) group (*p* < 0.05). # Represents statistical significance in relation to the CRS group (*p* < 0.05). One-way ANOVA followed by Bonferroni post hoc test.

**Figure 6:**
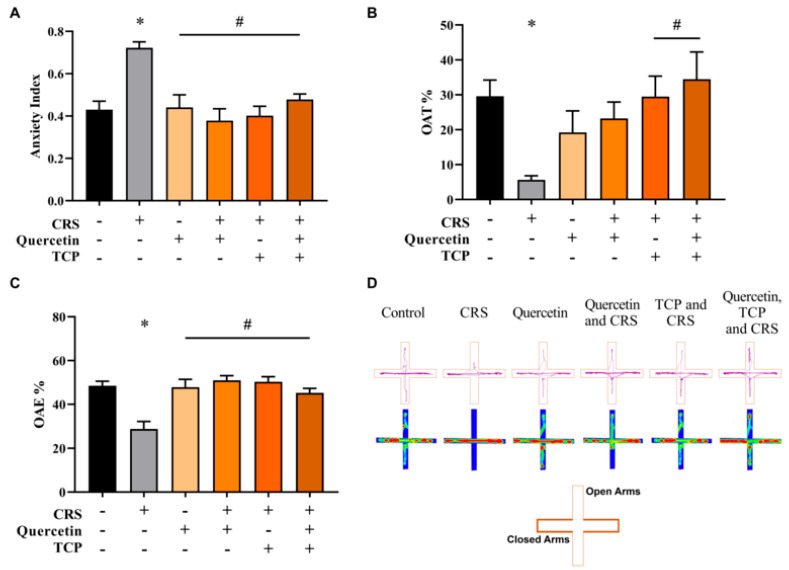
In vivo effects of treatments with Quercetin (50 mg/kg) and Tranylcypromine (10 mg/kg) in animals subjected to restraint stress in the elevated plus maze (EPM) test. (**A**) anxiety index. (**B**) percentage of time in open arms (OAT). (**C**) percentage of entries into open arms (OAE). (**D**) Representative tracking plots of an animal of each experimental group (first row). Heat map plots are representative of the mean of each group (second row). CRS = chronic restraint stress; TCP = tranylcypromine. Values are expressed as mean ± standard error of the mean (n = 8). * Represents statistical significance in relation to the control (non-stressed) group (*p* < 0.05). # Represents statistical significance in relation to the CRS group (*p* < 0.05). One-way ANOVA followed by Bonferroni post hoc test.

**Figure 7:**
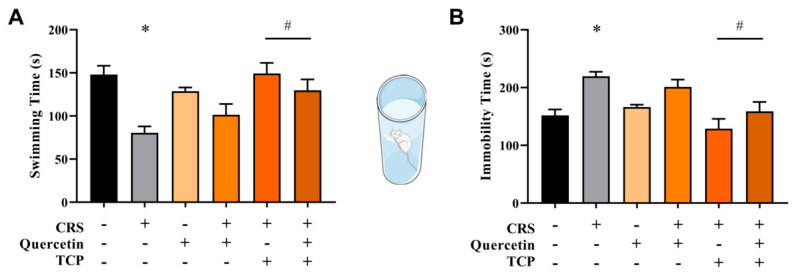
In vivo effects of treatments with Quercetin (50 mg/kg) and Tranylcypromine (10 mg/kg) in animals subjected to restraint stress in the forced swim test (FST). (**A**) Total swimming time. (**B**) Total immobility time. CRS = chronic restraint stress; TCP = tranylcypromine. Values are expressed as mean ± standard error of the mean (n = 8). * Represents statistical significance in relation to the control (non-stressed) group (*p* < 0.05). # Represents statistical significance in relation to the CRS group (*p* < 0.05). One-way ANOVA followed by Bonferroni post hoc test.

Alterations in mood and behavior are commonly associated with depression [2]. Therefore, a series of tasks were performed to evaluate behavioral changes in rats subjected to the CRS protocol. The following assessments were conducted: open field test (OFT) (Figure 4), novel object recognition (NOR) (Figure 5), elevated plus maze (EPM) (Figure 6), and forced swim test (FST) (Figure 7).

The outcomes of the OFT are displayed in Figure 4. There were no significant differences in the number of crossings in the central zone (F_5, 42_ = 1.19; *p* > 0.9999) (Figure 4A), grooming time (F_5, 42_ = 1.93; *p* = 0.7117) (Figure 4B), total distance (F_5, 42_ = 2.30; *p* = 0.7003) (Figure 4C), and number of rearings (F_5, 42_ = 2.33; *p* > 0.9999) (Figure 4E) between groups. However, the CRS group (subjected to stress) significantly increased the total number of fecal boli count during the behavioral test in comparison to control (non-stressed) animals (F_5, 42_ = 3.41; *p* = 0.0325) (Figure 4D). The group that received only quercetin, as well as the animals that were administered quercetin and subjected to the CRS model, demonstrated a reduction in this parameter in comparison to the CRS group (F_5, 42_ = 3.41; *p* = 0.0221 and *p* = 0.0325, respectively) (Figure 4D).

The novel object recognition (NOR) test was a two-step task conducted to assess the memory of animals (Figure 5). In the training (familiarization) session, animals explored two identical objects (familiar, F). During the test session, one of the familiar objects was replaced with a novel object (N). In the training session, there was no difference in the total time spent exploring the two identical objects (F_5, 82_ = 0.25; *p* = 0.9347) (Figure 5A). When the recognition index was calculated, it was observed that the animals from the CRS group, that did not receive any intervention, exhibited a significant decrease in this parameter compared to control (non-stressed) rats (F_5, 42_ = 9.19; *p* < 0.0001) (Figure 5B). Animals not subjected to stress that received quercetin administration (F_5, 42_ = 9.19; *p* < 0.0001) and animals subjected to the CRS protocol that received TCP alone or in association with quercetin administration significantly improved this parameter in comparison to the CRS group (F_5, 42_ = 9.19; *p* = 0.0269 and *p* = 0.0002, respectively) (Figure 5B).

Anxiety is often associated with depression [2]. Therefore, the elevated plus maze (EPM) test was performed over two consecutive days (training and test) to evaluate the anxiety-like behavior in animals (Figure 6). Rats of the CRS group that did not receive any intervention significantly decreased the percentage time spent in the open arms (OAT) (F_5, 42_ = 4.13; *p* = 0.0200) (Figure 6B) and the percentage of entries in the open arms (OAE) (F_5, 42_ = 9.97; *p* < 0.0001) (Figure 6C) in comparison to non-stressed animals (control group). Animals subjected to the CRS protocol but that received the administration of TCP alone and TCP in association with quercetin significantly increased the OAT in comparison to the CRS group (F_5, 42_ = 4.13; *p* = 0.0210 and *p* = 0.0030, respectively) (Figure 6B). Additionally, the percentage of OAE also significantly increased in animals of all groups in comparison to the CRS group (F_5, 42_ = 9.97; *p* = 0.0001, *p* < 0.0001, *p* < 0.0001 and *p* = 0.0009, respectively) (Figure 6C). Moreover, the anxiety index was determined by evaluating the OAT and OAE parameters (Figure 6A). The CRS group presented a significant increase in the anxiety index in comparison to the control group (non-stressed animals) (F_5, 42_ = 8.11; *p* = 0.0004). However, all groups significantly decreased this parameter in comparison to the CRS group (F_5, 42_ = 8.11; *p* = 0.0007, *p* < 0.0001, *p* < 0.0001 and *p* = 0.0042, respectively) (Figure 6A).

The forced swim test (FST) was conducted over two consecutive days, with one for training and another for testing, to evaluate depression-like behavior in rats. The results are presented in Figure 7. Figure 7A demonstrates that swimming duration decreased in animals subjected to the CRS model in comparison to the control group (F_5, 42_ = 6.82; *p* = 0.0006). However, animals subjected to the CRS protocol that received TCP or TCP in association with quercetin improved this parameter in comparison to animals of the CRS group (F_5, 42_ = 6.82; *p* = 0.0004 and *p* = 0.0249, respectively). Additionally, there was also an increase in the immobility time observed for CRS-challenged rats in comparison to the control group (F_5, 42_ = 7.40; *p* = 0.0051). However, animals subjected to the CRS protocol that received TCP or TCP in association with quercetin improved this parameter in comparison to animals of the CRS group (F_5, 42_ = 7.40; *p* < 0.0001 and *p* = 0.0167, respectively) (Figure 7B).

### 3.3. CRS-Induced Alterations in Cholinergic Parameters Are Reversed by Quercetin and TCP Treatments in the Brain of Male Rats

**Figure 8:**
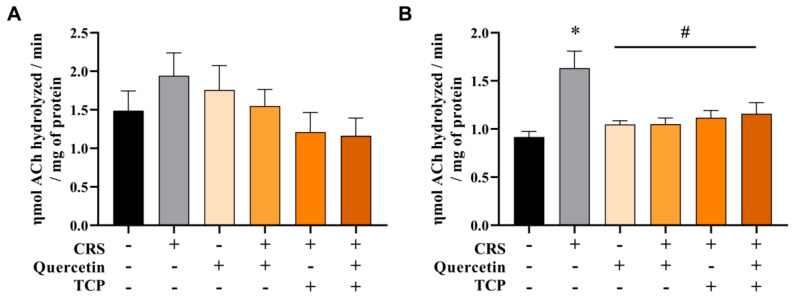
In vivo effects of treatments with Quercetin (50 mg/kg) and Tranylcypromine (10 mg/kg) in animals subjected to restraint stress on the activity of acetylcholinesterase (AChE) in the cerebral cortex (**A**) and hippocampus (**B**). CRS = chronic restraint stress; TCP = tranylcypromine. Values are expressed as mean ± standard error of the mean (n = 10). * Represents statistical significance in relation to the control (non-stressed) group (*p* < 0.05). # Represents statistical significance in relation to the CRS group (*p* < 0.05). One-way ANOVA followed by Bonferroni post hoc test.

**Figure 9:**
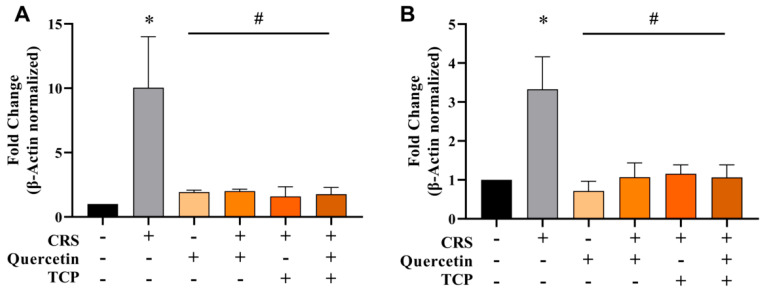
In vivo effects of treatments with Quercetin (50 mg/kg) and Tranylcypromine (10 mg/kg) in animals subjected to restraint stress on the gene expression profile of acetylcholinesterase (AChE) in cerebral cortex (**A**) and hippocampus (**B**). CRS = chronic restraint stress; TCP = tranylcypromine. Values are expressed as mean ± standard error of the mean (n = 3). * Represents statistical significance in relation to the control (non-stressed) group (*p* < 0.05). # Represents statistical significance in relation to the CRS group (*p* < 0.05). One-way ANOVA followed by Bonferroni post hoc test.

**Figure 10:**
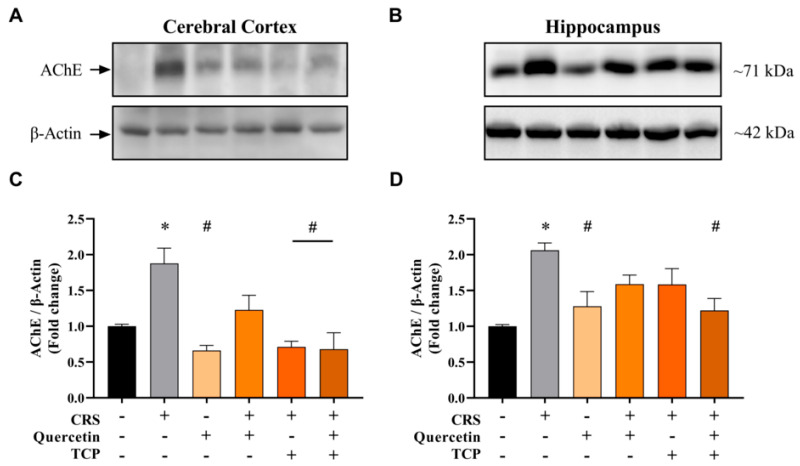
In vivo effects of treatments with Quercetin (50 mg/kg) and Tranylcypromine (10 mg/kg) in animals subjected to restraint stress on the density of acetylcholinesterase (AChE) in the cerebral cortex (**A**,**C**) and hippocampus (**B**,**D**). Representative Western blot bands (**A**,**B**). Bar graphs represent densitometric quantification of the Western blot bands normalized to the loading control (β-Actin) (**C**,**D**). CRS = chronic restraint stress; TCP = tranylcypromine. Values are expressed as mean ± standard error of the mean (n = 3). * Represents statistical significance in relation to the control (non-stressed) group (*p* < 0.05). # Represents statistical significance in relation to the CRS group (*p* < 0.05). One-way ANOVA followed by Bonferroni post hoc test.

Evidence has shown that chronic stress may alter cholinergic pathways in the brain [5]. Therefore, the impact of quercetin and TCP treatments on the modulation of AChE activity, expression, and protein levels in the cerebral cortex and hippocampus of male rats subjected to the CRS model was further examined (Figure 8, Figure 9 and Figure 10).

Results from the AChE activity in the cerebral cortex and hippocampus of male rats are presented in Figure 8. Findings demonstrate that AChE activity significantly increased in the hippocampus of animals of the CRS group in comparison to the control group (non-stressed animals) (F_5, 54_ = 6.29; *p* < 0.0001). However, animals subjected to the CRS protocol and treated with quercetin, TCP, and the association of TCP with quercetin significantly decreased AChE activity in relation to the CRS group (F_5, 54_ = 6.29; *p* = 0.0017, *p* = 0.0081, and *p* = 0.0199, respectively). The group treated with quercetin per se decreased AChE activity in relation to the CRS group (*p* = 0.0016) (Figure 8B). No significant differences between groups were noted for the AChE activity in the cerebral cortex (F_5, 54_ = 1.38; *p* = 0.8265) (Figure 8A).

To further examine the modulation of AChE in animals subjected to the CRS model and to assess the effects of quercetin and its association with TCP, gene expression analysis (Figure 9) and protein levels (Figure 10) were investigated. Gene expression analysis performed by qRT-PCR revealed a considerable increase in AChE mRNA levels in the cerebral cortex (F_5, 12_ = 4.27; *p* = 0.0223) (Figure 9A) and hippocampus (F_5, 12_ = 5.31; *p* = 0.0292) (Figure 9B) of male rats subjected to the CRS model in comparison to control (non-stressed) animals. Notably, all treatments decreased AChE mRNA levels in comparison to animals subjected to the CRS protocol in both brain structures, the cerebral cortex (F_5, 12_ = 4.27; *p* = 0.0436, *p* = 0.0457, *p* = 0.0340 and *p* = 0.0387, respectively) (Figure 9A) and hippocampus (F_5, 12_ = 5.31; *p* = 0.0123, *p* = 0.0362, *p* = 0.0470 and *p* = 0.0355, respectively) (Figure 9B).

The density of AChE was assessed in brain structures using Western blot analysis (Figure 10; Supplementary Materials). AChE levels were significantly higher in the cerebral cortex (F_5, 12_ = 8.92; *p* = 0.0194) (Figure 10A) and hippocampus (F_5, 12_ = 5.73; *p* = 0.0043) (Figure 10B) of CRS-challenged animals compared to the control group (non-stressed). The group treated with quercetin alone and not subjected to stress decreased AChE levels in comparison to animals from the CRS group in both brain structures, the cerebral cortex (*p* = 0.0016) (Figure 10A) and hippocampus (*p* = 0.0363) (Figure 10B). A reduction in AChE levels was detected in the cerebral cortex for the group treated with TCP and subjected to stress compared to CRS-challenged animals (*p* = 0.0023) (Figure 10A). Notably, the association of TCP and quercetin significantly decreased AChE protein levels in both brain structures, the cerebral cortex (F_5, 12_ = 8.92; *p* = 0.0019) (Figure 10A) and hippocampus (F_5, 12_ = 5.73; *p* = 0.0231) (Figure 10B), compared to the CRS group.

## 4. Discussion

MDD is a debilitating illness with a complex etiology. Despite significant progress in understanding its mechanisms and treatment interventions, new approaches are required to alleviate related symptoms [42]. To our knowledge, this study is the first to assess the effect of quercetin as a possible therapeutic strategy in association with TCP in a model of CRS in adult male rats. Findings demonstrated that this natural compound and its association with the drug were beneficial in improving behavioral phenotypes, memory, and cholinergic alterations induced by the CRS model in animals. We suggest that the prominent actions of quercetin in improving memory, and anxiety-like and depression-like behaviors can be partially explained by its anticholinergic mechanisms.

Chronic stress negatively impacts both mental and physical health and has been linked to the development of depression [5,7,43]. Several chronic stress models have been used to establish depression-like behaviors in rodents. The CRS model is an affordable and easy stress paradigm widely used in the literature for this purpose [8,44,45]. In our study, animals were subjected to a CRS model shown to recapitulate depression-like behaviors in rats [24,25]. Quercetin and TCP treatments, alone or in association, were used as interventions to reverse depression-like behaviors, assessed by a set of behavioral tasks.

The weight and water intake of animals were also measured throughout the experimental protocol. Although other research has highlighted a decrease in weight gain for male rats in a CRS model [46], only a trend towards a decrease in the weight of stressed animals was observed in our study. This asymmetry may reflect some habituation of male rats to the recurrent exposure to the stress model, which has been suggested by a recent study [47]. However, we did not investigate food intake in our study, which may have affected weight gain and metabolic rate. This point should be addressed by further investigations to give a clearer understanding about stress protocols to the weight of animals. In addition, the graphs of body weight and water consumption highlight some of the possible adverse effects of TCP. Treatment with quercetin, in turn, did not present side effects per se in these parameters, but it also did not modify the effects of TCP. The outcomes found in our study regarding TCP’s effects are in line with other research that has already described a decrease in food intake and body weight following chronic administration of TCP in male rats [48]. Literature data suggested that TCP may reduce rodent weight by increasing energy expenditure, decreasing food intake, and reducing fat content. These effects may be partially attributed to its pharmacological actions on neurotransmitter metabolism and other enzymatic targets related to fat regulation [49,50]. Nevertheless, future studies will aid to provide more insight into TCP’s effect on body weight and animal behavior.

Anhedonia is a common symptom of the human depression spectrum and is usually assessed by the SPT in rodents, where animals are given a choice between a sucrose solution and water [45]. Rodents prefer the sucrose solution, so a decrease in the intake of the sweet solution may reflect the anhedonia behavior [51]. In our study, a mild decrease in the sucrose preference was observed for stressed males compared to control (non-stressed) rats. These findings align with other studies that have shown no evident effects of chronic stress on sucrose preference [47]. Surprisingly, the groups of stressed males that received TCP administration, alone or in association with quercetin, reduced the preference for sucrose in the test. This outcome may not directly reflect the anhedonia behavior, but the decrease in general liquid intake, which may be another effect following TCP administration, such as other alterations found in our study and corroborated by previous literature [48]. However, more in-depth research needs to provide further clarification on this matter. It is noteworthy to comment that even though it is an important factor in the assessment of depression-like behavior, anhedonia behavior by the SPT is not always verified in chronic stress protocols, as many variables interfere in this aspect [45,52]. Other protocols for studying depression, such as the administration of corticosterone also do not usually alter the preference for sucrose in rodents [53]. Therefore, this result of our study should be evaluated in conjunction with the other behavioral tests performed.

Behavioral changes associated with the CRS model and potential beneficial effect of quercetin and TCP administration were further investigated. Regarding the exploratory behavior of animals assessed by the OFT, no significant alterations in the animals’ locomotor activity were found. However, although not significant, there was a mild reduction in the time spent grooming for the CRS group, which has been postulated previously as a depression-like behavior [44]. Interestingly, the fecal pellets produced by each animal significantly increased in the CRS group compared to the control. This higher defecation rate has been associated with greater emotionality or anxiety in the animals and is often a tool investigated in depression and anxiety models [54]. In fact, similarly to our study, research has shown that animals presented increased fecal pellet output during a stress protocol [46], suggesting that this may be a response that occurs during stressful events due to activation of colonic motility [55]. Nevertheless, quercetin per se and animals subjected to the stress protocol and that received quercetin significantly reversed this parameter compared to stressed males. Although not significant, a trend towards a decrease was also displayed for the groups subjected to stress that received TCP alone or in association with quercetin. Therefore, our findings suggest that quercetin may improve intestinal function and motility, a phenomenon already described in the literature [56].

The EPM test demonstrated the anxiety-like behavior present in the group subjected to restraint stress, given the increase in the anxiety index, which considers entries and time spent in the open arms of the apparatus. Although they are different conditions, anxiety is commonly observed as a symptom of depressive spectrum behavior [57]. In this scenario, both quercetin and TCP were able to normalize the anxiety index of animals in the EPM test, showing a promising anxiolytic effect for the natural molecule. There exist several supporting evidence showing that quercetin decreases anxiety-like behaviors in rodents. This flavonoid was shown to produce anxiolytic effects by the modulation of corticotrophin releasing factor (CRF) in mice [58] and to mitigate predator stress-induced anxiety-like behavior in rats [59]. Moreover, quercetin has been suggested to protect against the anxiogenic-like behavior displayed by cadmium exposure in rats [22] and against the anxiety-like behavior induced by streptozotocin (STZ) in a model of diabetes in rats [23]. Therefore, our results corroborate the anxiolytic-like effect of quercetin.

The FST is commonly used to assess depression-like symptoms in rodents and can be a powerful tool to screen for molecules with potential antidepressant properties [37]. This task demonstrated the effects of restraint stress, by decreasing the animals′ swimming time and increasing their immobility time in the apparatus. These alterations have been observed by several other studies that challenged rodents to stressful protocols [35,58,60,61,62], validating our experimental procedures. The administration with quercetin in CRS-challenged animals prevented significant variation in these parameters but was unable to restore to control levels. TCP treatment alone or in association with quercetin proved to be more efficient in restoring swimming and immobility parameters to control levels. Similarly to our study, the administration of TCP (10 mg/kg, oral gavage) significantly reduced the immobility time in a rodent depression model [12].

A growing body of evidence points to the effects of quercetin in mitigating depression-like behaviors. This flavonoid has been shown to reduce the immobility time in the FST in a model of corticotrophin releasing factor (CRF)-induced anxiety-like and depression-like behaviors [58]. In a protocol of corticosterone (CORT)-induced depression-like behavior, quercetin treatments (40 and 80 mg/kg) improved immobility and swimming times in comparison to the CORT group, likely by mechanisms involving the suppression of neuroinflammation and oxidative damage [61]. Additionally, in another model of chronic unpredictable mild stress (CUMS) in male rats, quercetin treatments (20, 40, and 60 mg/kg) showed antidepressant effect decreasing the immobility time in FST in a similar manner to the standard drug Fluoxetine (Flu) [62].

Cognitive deficits have been found in both MDD patients and animal models [63]. Thus, we investigated possible memory performance deficits associated with the CRS protocol in male rats. CRS significantly decreased the recognition index in the NOR test, which was reversed by the treatment with TCP alone or in association with quercetin in stress-challenged animals. Although the neurobiological mechanisms of TCP’s improvement in memory are not fully understood, we hypothesize that it can be related to its potential anti-inflammatory [11,12] and neuroprotective activities [64]. The beneficial effect of quercetin in improving memory in stress conditions has been suggested by some studies. Reports pointed out that quercetin improved memory deficits in mice challenged to a stress protocol by immobilization [60] and in rats subjected to chronic stress for 21 days [65].

Moreover, neuroprotective effects of quercetin on memory have also been reported by studies with animals exposed to cadmium [22] and diabetic rats [23]. In these studies, prevention of memory impairments by quercetin against the toxicity of cadmium and in STZ-induced diabetes was partially attributed to the improvement of acetylcholinesterase (AChE) activity in brain structures such as the cerebral cortex and hippocampus [22,23]. Thus, we hypothesize that cholinergic signaling may also play a role in the memory improvements demonstrated in our work.

Cholinergic system dysfunctions may represent likely contributors to the symptoms of MDD and have been associated with alterations in hippocampal function and consequent cognitive deficits [5]. Therefore, it has been postulated that acetylcholinesterase inhibitors (AChEIs) may offer some degree of antidepressant-like activity, but data are still conflicting in this regard [66,67,68]. Nevertheless, we demonstrated that AChE activity was increased in our CRS model compared to control males and this effect was reversed by quercetin treatment in the hippocampus. The stress-challenged animals that received TCP alone or in association with quercetin also reduced AChE activity compared to the CRS group in the hippocampus, but not in the cerebral cortex of rats, although a trend was identified. Divergent results from our findings regarding AChE activity have been previously reported [69], which may likely occur due to distinct chronic stress protocols.

The potential effect of quercetin in inhibiting AChE activity in brain structures has been documented in the literature. This natural compound decreased AChE activity in the cerebral cortex and hippocampus of STZ-diabetic rats [23], in cerebral cortex synaptosomes of rats exposed to cadmium [70], and in cerebral cortex synaptosomes of rats with hypothyroidism induced by methimazole (MMI) [71], and increased acetylcholine (Ach) levels in the brain of stressed mice [60]. These reports agree with our findings, and the possible mechanisms underlying this response may be due to the inhibitory effect of quercetin on AChE enzyme by its binding to the active site of the enzyme shown by in silico data [72,73]. Other data also suggested that the combination of quercetin and galantamine hydrobromide, an anticholinesterase and neurocognitive-enhancing drug commonly used for Alzheimer′s disease, has a synergistic inhibition effect on AChE [73].

Molecular assays were also conducted to provide more in-depth responses. AChE gene expression and protein density were significantly increased in the CRS protocol in both brain structures. Overall, quercetin and TCP proved to reverse CRS-induced alterations in the AChE enzyme. There is little information about TCP’s anticholinesterase action, although this possibility has been suggested previously [74]. In this sense, this field provides an opportunity for future research. Regarding the effects of quercetin on AChE demonstrated by enzymatic and molecular analyses, these may help to partially explain the memory improvement shown by the NOR test conducted in our study, especially considering the outcomes for the hippocampus, a brain region critical for memory and cognitive functions [5]. Thus, our findings help to provide some understanding into the potential therapeutic-like effects of quercetin as an anticholinesterase molecule, especially in association with an antidepressant drug.

Despite the limitations of animal models, the results obtained in the behavioral tasks conducted in our study suggest that the CRS model effectively induced stable behavioral changes shown to recapitulate depression-like and anxiety-like behaviors in rats. However, it is noteworthy to comment that the divergences in the results of behavioral tasks between our study and others may indicate some variations in the experimental protocols, which should be considered by future studies. There are also constraints associated with the present study that should be mentioned. The levels of corticosterone and monoamines in the brain structures were not investigated and remain a topic for future investigation. Inflammatory pathways also remain to be explored to provide insights into the mechanisms in brain regions in depression. This study only used male rats, so it is important for future investigations to include females to address possible sex-related outcomes. The food consumption was also not assessed, which may have provided more information about the stress-related findings for our protocol and the effects of treatments. Moreover, future research should explore the role of the microbiota-gut–brain axis in depression, as it represents a promising field of study.

## 5. Conclusions

Altogether, the findings presented here suggest the protective role of quercetin in ameliorating CRS-induced alterations in male rats. This flavonoid improved memory, anxiety-like, and depression-like behaviors in rodents, possibly by improving cholinergic functions. Therefore, quercetin was shown to present therapeutic-like effects, supporting its action as a promising agent especially in association with the standard medication for depression. Despite the complexity of MDD and the intricate mechanisms underlying its pathophysiology, the data of this study provide a scientific foundation for the potential protective effects of quercetin and its synergistic action with the drug TCP. However, future research should be conducted to provide further clarification into the mechanisms of this natural compound and the development of new therapeutic strategies for depression.

## Data Availability

All data are provided within the manuscript or its Supplementary Materials.

## Supplementary Materials

The following supporting information can be downloaded at: https://www.mdpi.com/article/10.3390/brainsci15070709/s1. Western blot membranes.

## References

[1] World Health Organization (WHO) Depressive Disorder (Depression). https://www.who.int/news-room/fact-sheets/detail/depression.

[2] Kennedy S.H. (2008). Core symptoms of major depressive disorder: Relevance to diagnosis and treatment. Dialogues Clin. Neurosci..

[3] Cui L., Li S., Wang S., Wu X., Liu Y., Yu W., Wang Y., Tang Y., Xia M., Li B. (2024). Major depressive disorder: Hypothesis, mechanism, prevention and treatment. Signal Transduct. Target. Ther..

[4] Villas Boas G.R., Boerngen de Lacerda R., Paes M.M., Gubert P., Almeida W.L.D.C., Rescia V.C., de Carvalho P.M.G., de Carvalho A.A.V., Oesterreich S.A. (2019). Molecular aspects of depression: A review from neurobiology to treatment. Eur. J. Pharmacol..

[5] Dagytė G., Den Boer J.A., Trentani A. (2011). The cholinergic system and depression. Behav. Brain Res..

[6] Dulawa S.C., Janowsky D.S. (2019). Cholinergic regulation of mood: From basic and clinical studies to emerging therapeutics. Mol. Psychiatry.

[7] Hassamal S. (2023). Chronic stress, neuroinflammation, and depression: An overview of pathophysiological mechanisms and emerging anti-inflammatories. Front. Psychiatry.

[8] Thakare V.N., Dhakane V.D., Patel B.M. (2017). Attenuation of acute restraint stress-induced depressive like behavior and hippocampal alterations with protocatechuic acid treatment in mice. Metab. Brain Dis..

[9] Delgado P.L. (2000). Depression: The case for a monoamine deficiency. J. Clin. Psychiatry.

[10] Ramaholimihaso T., Bouazzaoui F., Kaladjian A. (2020). Curcumin in Depression: Potential Mechanisms of Action and Current Evidence-A Narrative Review. Front. Psychiatry.

[11] Park H., Han K.M., Jeon H., Lee J.S., Lee H., Jeon S.G., Park J.H., Kim Y.G., Lin Y., Lee Y.H. (2020). The MAO Inhibitor Tranylcypromine Alters LPS- and Aβ-Mediated Neuroinflammatory Responses in Wild-type Mice and a Mouse Model of AD. Cells.

[12] Tomaz V.S., Chaves Filho A.J.M., Cordeiro R.C., Jucá P.M., Soares M.V.R., Barroso P.N., Cristino L.M.F., Jiang W., Teixeira A.L., de Lucena D.F. (2020). Antidepressants of different classes cause distinct behavioral and brain pro- and anti-inflammatory changes in mice submitted to an inflammatory model of depression. J. Affect. Disord..

[13] Hwang J.S., Lee S.A., Hong S.S., Lee K.S., Lee M.K., Hwang B.Y., Ro J.S. (2005). Monoamine oxidase inhibitory components from the roots of *Sophora flavescens*. Arch. Pharm. Res..

[14] Han X.H., Hong S.S., Hwang J.S., Lee M.K., Hwang B.Y., Ro J.S. (2007). Monoamine oxidase inhibitory components from *Cayratia japonica*. Arch. Pharm. Res..

[15] Saaby L., Rasmussen H.B., Jäger A.K. (2009). MAO-A inhibitory activity of quercetin from *Calluna vulgaris* (L.) Hull. J. Ethnopharmacol..

[16] Yoshino S., Hara A., Sakakibara H., Kawabata K., Tokumura A., Ishisaka A., Kawai Y., Terao J. (2011). Effect of quercetin and glucuronide metabolites on the monoamine oxidase-A reaction in mouse brain mitochondria. Nutrition.

[17] Bandaruk Y., Mukai R., Kawamura T., Nemoto H., Terao J. (2012). Evaluation of the inhibitory effects of quercetin-related flavonoids and tea catechins on the monoamine oxidase-A reaction in mouse brain mitochondria. J. Agric. Food Chem..

[18] Bhagwat S., Haytowitz D.B., Holden J.M. (2011). USDA Database for the Flavonoid Content of Selected Foods.

[19] Silvestro S., Bramanti P., Mazzon E. (2021). Role of Quercetin in Depressive-Like Behaviors: Findings from Animal Models. Appl. Sci..

[20] Wang G., Wang Y., Yao L., Gu W., Zhao S., Shen Z., Lin Z., Liu W., Yan T. (2022). Pharmacological Activity of Quercetin: An Updated Review. Evid. Based Complement. Altern. Med..

[21] Chen S., Tang Y., Gao Y., Nie K., Wang H., Su H., Wang Z., Lu F., Huang W., Dong H. (2022). Antidepressant Potential of Quercetin and its Glycoside Derivatives: A Comprehensive Review and Update. Front. Pharmacol..

[22] Abdalla F.H., Schmatz R., Cardoso A.M., Carvalho F.B., Baldissarelli J., de Oliveira J.S., Rosa M.M., Gonçalves Nunes M.A., Rubin M.A., da Cruz I.B. (2014). Quercetin protects the impairment of memory and anxiogenic-like behavior in rats exposed to cadmium: Possible involvement of the acetylcholinesterase and Na(+),K(+)-ATPase activities. Physiol. Behav..

[23] Maciel R.M., Carvalho F.B., Olabiyi A.A., Schmatz R., Gutierres J.M., Stefanello N., Zanini D., Rosa M.M., Andrade C.M., Rubin M.A. (2016). Neuroprotective effects of quercetin on memory and anxiogenic-like behavior in diabetic rats: Role of ectonucleotidases and acetylcholinesterase activities. Biomed. Pharmacother..

[24] Cunha G.M., Canas P.M., Oliveira C.R., Cunha R.A. (2006). Increased density and synapto-protective effect of adenosine A2A receptors upon sub-chronic restraint stress. Neuroscience.

[25] Dias L., Lopes C.R., Gonçalves F.Q., Nunes A., Pochmann D., Machado N.J., Tomé A.R., Agostinho P., Cunha R.A. (2021). Crosstalk Between ATP-P2X7 and Adenosine A2A Receptors Controlling Neuroinflammation in Rats Subject to Repeated Restraint Stress. Front. Cell. Neurosci..

[26] Pereira Braga C., Momentti A.C., Barbosa Peixoto F., de Fátima Ferreira Baptista R., dos Santos F.A., Fava F.H., Fernandes A.A. (2013). Influence of treatment with quercetin on lipid parameters and oxidative stress of pregnant diabetic rats. Can. J. Physiol. Pharmacol..

[27] De Mattos B.D.S., Soares M.S.P., Spohr L., Pedra N.S., Teixeira F.C., de Souza A.A., Stefanello F.M., Baldissarelli J., Gamaro G.D., Spanevello R.M. (2020). Quercetin prevents alterations of behavioral parameters, delta-aminolevulinic dehydratase activity, and oxidative damage in brain of rats in a prenatal model of autism. Int. J. Dev. Neurosci..

[28] Gorbenko N.I., Borikov O.Y., Kiprych T.V., Ivanova O.V., Taran K.V., Litvinova T.S. (2021). Quercetin improves myocardial redox status in rats with type 2 diabetes. Endocr. Regul..

[29] Greenshaw A.J., Nazarali A.J., Rao T.S., Baker G.B., Coutts R.T. (1988). Chronic tranylcypromine treatment induces functional alpha 2-adrenoceptor down-regulation in rats. Eur. J. Pharmacol..

[30] Malyszko J., Urano T., Takada Y., Takada A. (1994). Serotonergic systems in brain and blood under stress and tranylcypromine treatment in rats. Brain Res. Bull..

[31] Castro M.F.V., Assmann C.E., Stefanello N., Reichert K.P., Palma T.V., da Silva A.D., Miron V.V., Mostardeiro V.B., Morsch V.M.M., Schetinger M.R.C. (2023). Caffeic acid attenuates neuroinflammation and cognitive impairment in streptozotocin-induced diabetic rats: Pivotal role of the cholinergic and purinergic signaling pathways. J. Nutr. Biochem..

[32] Carvalho F.B., Gutierres J.M., Bueno A., Agostinho P., Zago A.M., Vieira J., Frühauf P., Cechella J.L., Nogueira C.W., Oliveira S.M. (2017). Anthocyanins control neuroinflammation and consequent memory dysfunction in mice exposed to lipopolysaccharide. Mol. Neurobiol..

[33] Miron V.V., Assmann C.E., Mostardeiro V.B., da Silveira M.V., Copetti P.M., Bissacotti B.F., Schirmann A.A., Castro M.F.V., Gutierres J.M., da Cruz Fernandes M. (2024). Neuroprotective effect of long-term resistance physical exercise against memory damage elicited by a lipopolysaccharide-induced neuroinflammation model in male rats. J. Neurosci. Res..

[34] Pellow S., Chopin P., File S.E., Briley M. (1985). Validation of open:closed arm entries in an elevated plus-maze as a measure of anxiety in the rat. J. Neurosci. Methods.

[35] Jung J.T.K., Marques L.S., Zborowski V.A., Silva G.L., Nogueira C.W., Zeni G. (2023). Resistance Training Modulates Hippocampal Neuroinflammation and Protects Anxiety-Depression-like Dyad Induced by an Emotional Single Prolonged Stress Model. Mol. Neurobiol..

[36] Porsolt R.D., Le Pichon M., Jalfre M. (1977). Depression: A new animal model sensitive to antidepressant treatments. Nature.

[37] Yankelevitch-Yahav R., Franko M., Huly A., Doron R. (2015). The forced swim test as a model of depressive-like behavior. J. Vis. Exp..

[38] Ellman G.l., Courtney K.d., Andres V., Feather-Stone R.M. (1961). A new and rapid colorimetric determination of acetylcholinesterase activity. Biochem. Pharmacol..

[39] Bradford M.M. (1976). A rapid and sensitive method for the quantitation of microgram quantities of protein utilizing the principle of protein-dye binding. Anal. Biochem..

[40] Assmann C.E., Mostardeiro V.B., Weis G.C.C., Reichert K.P., de Oliveira Alves A., Miron V.V., Bagatini M.D., Palma T.V., de Andrade C.M., Pillat M.M. (2021). Aluminum-Induced Alterations in Purinergic System Parameters of BV-2 Brain Microglial Cells. J. Immunol. Res..

[41] Rebola N., Simões A.P., Canas P.M., Tomé A.R., Andrade G.M., Barry C.E., Agostinho P.M., Lynch M.A., Cunha R.A. (2011). Adenosine A2A receptors control neuroinflammation and consequent hippocampal neuronal dysfunction. J. Neurochem..

[42] Tian H., Hu Z., Xu J., Wang C. (2022). The molecular pathophysiology of depression and the new therapeutics. MedComm.

[43] James K.A., Stromin J.I., Steenkamp N., Combrinck M.I. (2023). Understanding the relationships between physiological and psychosocial stress, cortisol and cognition. Front. Endocrinol..

[44] Seewoo B.J., Hennessy L.A., Feindel K.W., Etherington S.J., Croarkin P.E., Rodger J. (2020). Validation of Chronic Restraint Stress Model in Young Adult Rats for the Study of Depression Using Longitudinal Multimodal MR Imaging. eNeuro.

[45] Mao Y., Xu Y., Yuan X. (2022). Validity of chronic restraint stress for modeling anhedonic-like behavior in rodents: A systematic review and meta-analysis. J. Int. Med. Res..

[46] Olave F.A., Aguayo F.I., Román-Albasini L., Corrales W.A., Silva J.P., González P.I., Lagos S., García M.A., Alarcón-Mardones M., Rojas P.S. (2022). Chronic restraint stress produces sex-specific behavioral and molecular outcomes in the dorsal and ventral rat hippocampus. Neurobiol. Stress.

[47] Pansarim V., Leite-Panissi C.R.A., Schmidt A. (2023). Chronic restraint stress alters rat behavior depending on sex and duration of stress. Behav. Processes.

[48] Assareh N., ElBatsh M.M., Marsden C.A., Kendall D.A. (2012). The effects of chronic administration of tranylcypromine and rimonabant on behaviour and protein expression in brain regions of the rat. Pharmacol. Biochem. Behav..

[49] Shemesh A., Abdulla A., Yang F., Chua S.C., Pessin J.E., Zong H. (2014). The antidepressant trans-2-phenylcyclopropylamine protects mice from high-fat-diet-induced obesity. PLoS ONE.

[50] Carpéné C., Boulet N., Chaplin A., Mercader J. (2019). Past, Present and Future Anti-Obesity Effects of Flavin-Containing and/or Copper-Containing Amine Oxidase Inhibitors. Medicines.

[51] Slattery D.A., Cryan J.F. (2017). Modelling depression in animals: At the interface of reward and stress pathways. Psychopharmacology.

[52] Markov D.D. (2022). Sucrose Preference Test as a Measure of Anhedonic Behavior in a Chronic Unpredictable Mild Stress Model of Depression: Outstanding Issues. Brain Sci..

[53] Berger S., Gureczny S., Reisinger S.N., Horvath O., Pollak D.D. (2019). Effect of Chronic Corticosterone Treatment on Depression-Like Behavior and Sociability in Female and Male C57BL/6N Mice. Cells.

[54] Seibenhener M.L., Wooten M.C. (2015). Use of the Open Field Maze to measure locomotor and anxiety-like behavior in mice. J. Vis. Exp..

[55] Taché Y., Million M. (2015). Role of Corticotropin-releasing Factor Signaling in Stress-related Alterations of Colonic Motility and Hyperalgesia. J. Neurogastroenterol. Motil..

[56] Di Carlo G., Mascolo N., Izzo A.A., Capasso F., Autore G. (1994). Effects of quercetin on the gastrointestinal tract in rats and mice. Phytother. Res..

[57] Ausderau K.K., Colman R.J., Kabakov S., Schultz-Darken N., Emborg M.E. (2023). Evaluating depression- and anxiety-like behaviors in non-human primates. Front. Behav. Neurosci..

[58] Bhutada P., Mundhada Y., Bansod K., Ubgade A., Quazi M., Umathe S., Mundhada D. (2010). Reversal by quercetin of corticotrophin releasing factor induced anxiety- and depression-like effect in mice. Prog. Neuropsychopharmacol. Biol. Psychiatry.

[59] Toumi M.L., Merzoug S., Baudin B., Tahraoui A. (2013). Quercetin alleviates predator stress-induced anxiety-like and brain oxidative signs in pregnant rats and immune count disturbance in their offspring. Pharmacol. Biochem. Behav..

[60] Samad N., Saleem A., Yasmin F., Shehzad M.A. (2018). Quercetin protects against stress-induced anxiety- and depression-like behavior and improves memory in male mice. Physiol. Res..

[61] Ge C., Wang S., Wu X., Lei L. (2023). Quercetin mitigates depression-like behavior via the suppression of neuroinflammation and oxidative damage in corticosterone-induced mice. J. Chem. Neuroanat..

[62] Wang M., Wei X., Jia Y., Wang C., Wang X., Zhang X., Li D., Wang Y., Gao Y. (2024). Quercetin alleviates chronic unpredictable mild stress-induced depression-like behavior by inhibiting NMDAR1 with α2δ-1 in rats. CNS Neurosci. Ther..

[63] Wu A., Zhang J. (2023). Neuroinflammation, memory, and depression: New approaches to hippocampal neurogenesis. J. Neuroinflamm..

[64] Caraci F., Pappalardo G., Basile L., Giuffrida A., Copani A., Tosto R., Sinopoli A., Giuffrida M.L., Pirrone E., Drago F. (2015). Neuroprotective effects of the monoamine oxidase inhibitor tranylcypromine and its amide derivatives against Aβ(1-42)-induced toxicity. Eur. J. Pharmacol..

[65] Mohammadi H.S., Goudarzi I., Lashkarbolouki T., Abrari K., Elahdadi Salmani M. (2014). Chronic administration of quercetin prevent spatial learning and memory deficits provoked by chronic stress in rats. Behav. Brain Res..

[66] Fitzgerald P.J., Hale P.J., Ghimire A., Watson B.O. (2020). The cholinesterase inhibitor donepezil has antidepressant-like properties in the mouse forced swim test. Transl. Psychiatry.

[67] Fitzgerald P.J., Hale P.J., Ghimire A., Watson B.O. (2021). Multiple cholinesterase inhibitors have antidepressant-like properties in the mouse forced swim test. Behav. Brain Res..

[68] Fitzgerald P.J., Hale P.J., Ghimire A., Watson B.O. (2021). Repurposing Cholinesterase Inhibitors as Antidepressants? Dose and Stress-Sensitivity May Be Critical to Opening Possibilities. Front. Behav. Neurosci..

[69] Srikumar B.N., Raju T.R., Shankaranarayana Rao B.S. (2006). The involvement of cholinergic and noradrenergic systems in behavioral recovery following oxotremorine treatment to chronically stressed rats. Neuroscience.

[70] Abdalla F.H., Cardoso A.M., Pereira L.B., Schmatz R., Gonçalves J.F., Stefanello N., Fiorenza A.M., Gutierres J.M., Serres J.D., Zanini D. (2013). Neuroprotective effect of quercetin in ectoenzymes and acetylcholinesterase activities in cerebral cortex synaptosomes of cadmium-exposed rats. Mol. Cell. Biochem..

[71] Baldissarelli J., Santi A., Schmatz R., Abdalla F.H., Cardoso A.M., Martins C.C., Dias G.R., Calgaroto N.S., Pelinson L.P., Reichert K.P. (2017). Hypothyroidism Enhanced Ectonucleotidases and Acetylcholinesterase Activities in Rat Synaptosomes can be Prevented by the Naturally Occurring Polyphenol Quercetin. Cell Mol. Neurobiol..

[72] Islam M.R., Zaman A., Jahan I., Chakravorty R., Chakraborty S. (2013). In silico QSAR analysis of quercetin reveals its potential as therapeutic drug for Alzheimer’s disease. J. Young Pharm..

[73] Liao Y., Mai X., Wu X., Hu X., Luo X., Zhang G. (2022). Exploring the Inhibition of Quercetin on Acetylcholinesterase by Multispectroscopic and In Silico Approaches and Evaluation of Its Neuroprotective Effects on PC12 Cells. Molecules.

[74] Fortes Z.B., Reis C.C., Scivoletto R. (1980). Anticholinesterase activity of tranylcypromine and its isomers. Rev. Bras. Pesqui. Med. Biol..

