# Supplementary material for: Quercetin and Tranylcypromine Improve Memory, Behavioral Performance, and Cholinergic Function in Male Rats Subjected to Chronic Restraint Stress
Source: Brain Sci. 2025 Jul 1;15(7):709. doi: 10.3390/brainsci15070709 (PMC12293918; doi:10.3390/brainsci15070709)
Supplement: Supplementary file 1 [file brainsci-15-00709-s001.zip › brainsci-3709704-supplementary.pdf]

Antibody Anti-	Repeat 1	Repeat 2	Repeat 3
AChE<br>71 kDa<br>Cerebral Cortex	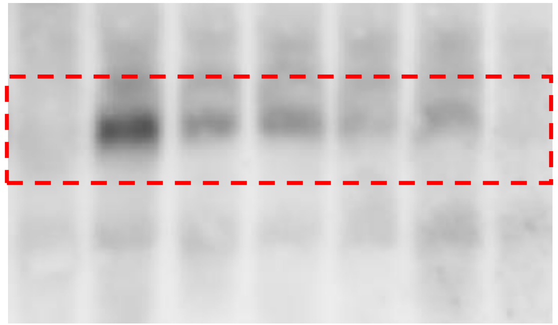	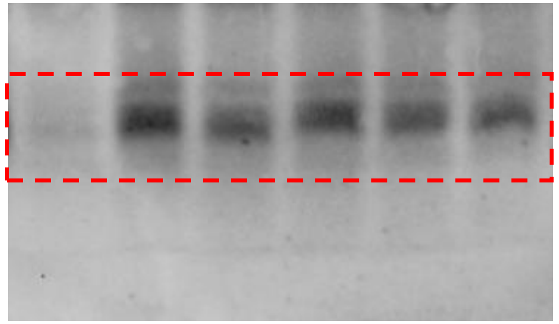	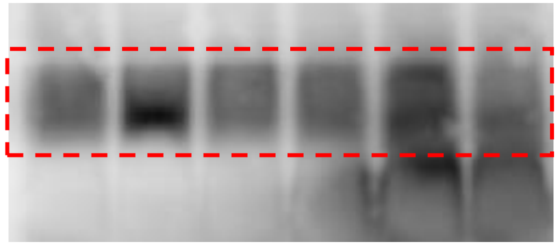
AChE<br>71 kDa<br>Hippocampus	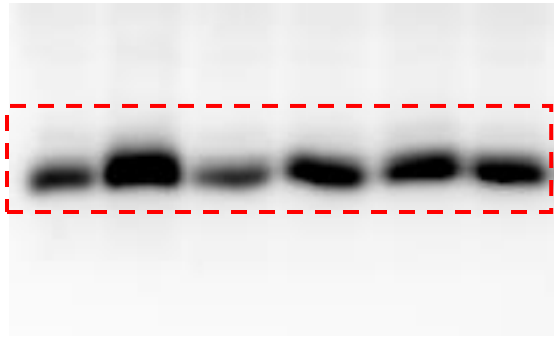	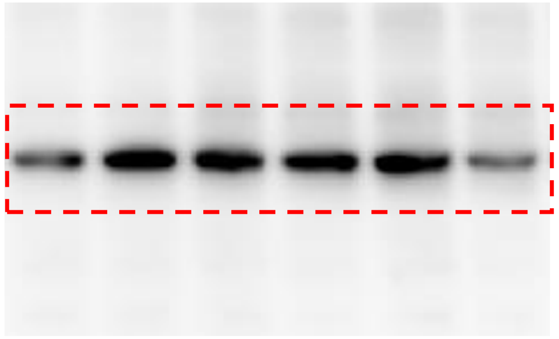	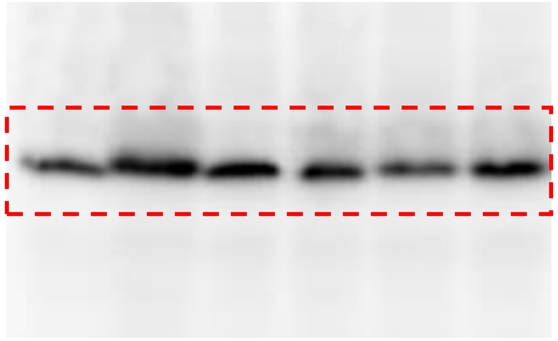

Antibody Anti-	Repeat 1	Repeat 2	Repeat 3
β-Actin<br>42 kDa<br>Cerebral Cortex	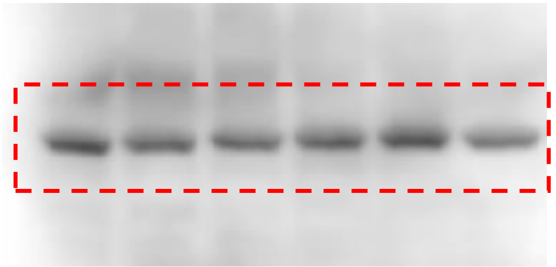	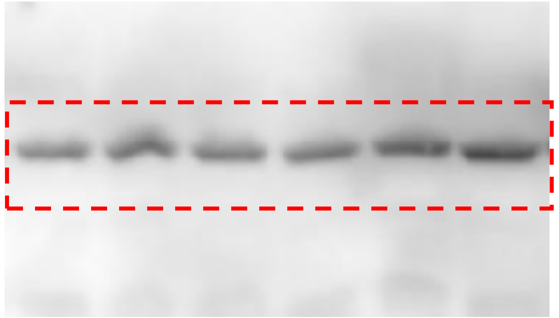	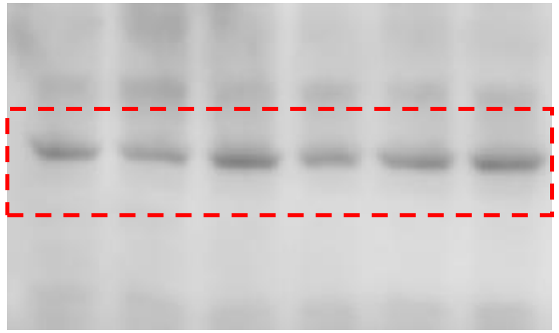
β-Actin<br>42 kDa<br>Hippocampus	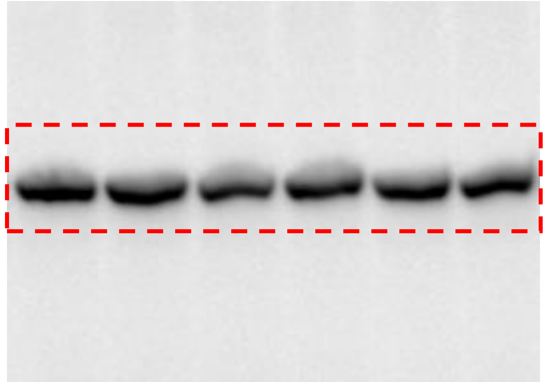	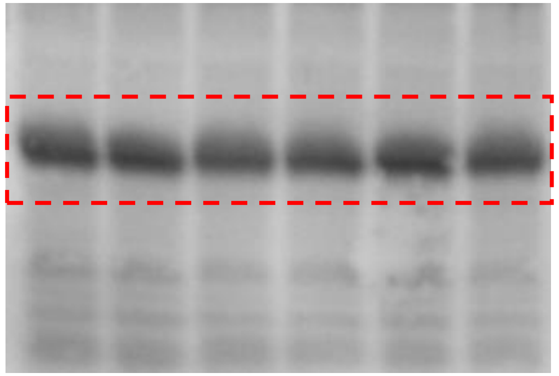	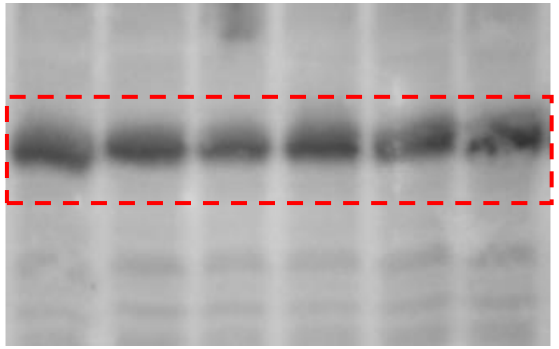
